# Role of* luxS* in Stress Tolerance and Adhesion Ability in* Lactobacillus plantarum* KLDS1.0391

**DOI:** 10.1155/2018/4506829

**Published:** 2018-01-30

**Authors:** Fang-Fang Jia, Hui-Qi Zheng, Si-Rui Sun, Xue-Hui Pang, Yu Liang, Jia-Cui Shang, Zong-Tao Zhu, Xiang-Chen Meng

**Affiliations:** Key Laboratory of Dairy Science, Ministry of Education, Northeast Agricultural University, Harbin 150030, China

## Abstract

*Lactobacillus plantarum*, a probiotic, has a high survival rate and high colonization ability in the gastrointestinal tract. Tolerance to the gastrointestinal environment and adhesion to intestinal epithelial cells by some* Lactobacillus *species (excluding* L. plantarum*) are related to* luxS*/AI-2. Here, the role of* luxS* in tolerance to simulated digestive juice (SDJ) and adhesion to Caco-2 cells by* L. plantarum *KLDS1.0391 (hereafter, KLDS1.0391) was investigated. The KLDS1.0391* luxS* mutant strain was constructed by homologous recombination. When* luxS *was deleted, acid and bile salt tolerance and survival rates in SDJ significantly decreased (*p* < 0.05 for all). The ability of the* luxS* deletion strain to adhere to Caco-2 cells was markedly lower than that of the wild-type strain (*p* < 0.05). The ability of the* luxS* mutant strain to adhere (competition, exclusion, and displacement) to* Escherichia coli* ATCC 25922 was significantly lower than that of the wild-type strain (*p* < 0.05 for all). A significant decrease was noted only in the exclusion adhesion inhibition of the* luxS* mutant strain to* Salmonella typhimurium* ATCC 14028 (*p* < 0.05). These results indicate that the* luxS* gene plays an important role in the gastrointestinal environment tolerance and adhesion ability of KLDS1.0391.

## 1. Introduction

*Lactobacillus* belongs to the large group of Lactic Acid Bacteria (LAB), which is comprised of more than 150 different species [1].* Lactobacillus plantarum* is one of the most widespread* Lactobacillus* species and is commonly found in starchy food, cereals, meat, dairy products, vegetables, fruits, and beverages [2]. Some strains that are part of the microbial flora of the host provide health benefits to the host [3]. For instance, intestinal barrier function is enhanced and irritable bowel syndrome symptoms decrease after* L. plantarum *intake [4, 5]. In addition, the general health status of elderly people improves after consumption of this probiotic [6].* L. plantarum* is considered an important industrial microbe [7–9] and has been widely utilized in food industries via food-related technologies [10, 11].

According to Food and Agriculture Organization (FAO)/World Health Organization (WHO) regulations (2002), the primary criterion for probiotics is that they must have high tolerance to the gastrointestinal tract (GIT) environment and high adhesion ability, which enables their beneficial effects, that is, maintenance of the balance of intestinal flora [12]. Currently, consumption of probiotic-containing food is the common approach for its transit to the GIT. In the GIT, the probiotics are subjected to various intestinal environment stresses, such as acid and bile salt.* L. plantarum* can ferment various kinds of carbohydrates; for example, it can ferment hexoses to d- and l-lactic acids via enzymes and metabolic pathways or pentoses to lactic acid and acetic acid in the presence of phospho-acetylase [10]. Thus,* L. plantarum* has excellent acid tolerance and may therefore have a high survival rate in the GIT and high colonization ability in the intestinal tract of humans and other mammals, being a part of the microbial flora in these habitats.

The genes encoding stress-related and adhesion-related proteins in the* L. plantarum* genome can provide more information regarding the high tolerance and adhesion of* L. plantarum* [13–16]. Some studies have shown that the* luxS* gene or autoinducer-2 (AI-2) signal molecule is associated with acid tolerance [17–19] and the ability to adhere to intestinal epidermal cells in* L. acidophilus* and* L. rhamnosus* [20, 21]. Moreover, the* luxS *gene encodes the enzyme* S*-ribosylhomocysteine, which catalyzes a reaction involved in AI-2 synthesis in the activated methyl cycle [22]. The* luxS* gene sequences are conserved across various microbial species [23, 24], and the gene is found in more than 80 kinds of bacteria, including not only pathogenic bacteria but also many probiotics like* Bifidobacterium *[25] and* Lactobacillus *[17]. Although the* luxS *gene is related to acid tolerance of and adhesion by some* Lactobacillus *species [26], for example,* L. acidophilus* and* L. rhamnosus*, the relationship between the* luxS* gene and bile salt tolerance of* Lactobacillus* has rarely been studied. In particular, the role of* luxS* in resistance to acid and bile salt stresses and in the adhesion ability of* L. plantarum *is still not clear and is being studied.

KLDS1.0391 had been isolated earlier from a traditional dairy product named “jiaoke,” obtained from Inner Mongolia [27]. In the current study, bioinformatics analysis of the LuxS protein of KLDS1.0391 was performed. Afterwards, we analyzed the effect of the* luxS* gene on the tolerance of KLDS1.0391 to the GIT environment and the adhesion properties of the strain, by constructing a* luxS* gene mutant.

## 2. Materials and Methods

### 2.1. Bacterial Strains and Culture Conditions

KLDS1.0391, which was preserved at the Key Laboratory of Dairy Science-Dairy Industrial Culture Collection, was routinely grown in de Man-Rogosa-Sharpe (MRS) broth at 37°C. Chloramphenicol (Cm; 4.0 *μ*g/mL; Sigma, USA) was added to the MRS broth for selection and propagation of the* luxS* mutant strain of KLDS1.0391.* Vibrio harveyi* BB170 and* V. harveyi* BB120, which were purchased from American Type Culture Collection, were grown in modified autoinducer bioassay (AB) broth at 30°C. AB broth (1 L) was sterilized at 121°C for 20 min, following which 50% glycerine (20 mL), arginine (10 mL), and potassium phosphate buffer (10 mL, 1.0 mol/L, pH 7.0) that had been filtered through a 0.22-*μ*m filter were added to AB broth.* Escherichia coli* ATCC 25922 and* Salmonella typhimurium *ATCC 14028 were obtained from the Institute of Microbiology, Heilongjiang Academy of Sciences (China), and the National Institutes for Food and Drug Control (NIFDC, China), respectively. Both were grown in nutrient broth at 37°C.

### 2.2. Bioinformatics Analysis of LuxS Protein

Multiple-sequence alignment of LuxS protein was generated using ClustalX6. The phylogeny tree of LuxS protein was constructed using the neighbor-joining method with MEGA 6. Basic property analysis and functional predictions (including transmembrane domains, localization sites, tertiary structures, and conserved domains) for LuxS proteins were performed using Expasy (https://www.expasy.ch/tools/protparam.html), TMHMM (http://www.cbs.dtu.dk/services/TMHMM/), PSORT (http://psort.nibb.ac.jp), SWISS-MODEL (http://swissmodel.expasy.org/workspace/index.php?func=modelling_%20simple1), and NetPhos (http://www.cbs.dtu.dk/services/NetPhos/) [28]. The related data and sequences of LuxS protein were retrieved from GenBank (https://www.ncbi.nlm.nih.gov/genbank/).

### 2.3. Construction of the KLDS1.0391* luxS* Mutant Strain

The* luxS* gene was knocked out in KLDS1.0391 by homologous recombination. Flanking regions of the* luxS* gene (upstream and downstream) were amplified from the KLDS1.0391 DNA template. These two fragments were connected to the corresponding restriction sites of the pNZ5319 plasmid, which is a Cm-resistant derivative of the pACYC184 plasmid. Details regarding the construction procedure of the homologous recombination vector are shown in Figure S1. The resulting homologous recombination vector pNZ5319-luxS was electrotransformed into the competent cells of KLDS1.0391 according to the method proposed by Landete et al. [29], with some modifications. After incubation for 3 days at 37°C on MRS agar containing Cm (4.0 *μ*g/mL), the successfully electrotransformed cells of KLDS1.0391 were identified by polymerase chain reaction (PCR). The* luxS *mutant strain was selected by plating onto MRS agar with Cm and identification by PCR. The reaction mixture (50 *μ*L) contained 35.5 *μ*L ddH_2_O, 5.0 *μ*L 10x Taq DNA polymerase buffer, 4 *μ*L dNTPs (2.5 mmol/L), 2.0 *μ*L PCR forward primer (10 mM), 2.0 *μ*L PCR reverse primer (10 mM), 1.0 *μ*L DNA sample, and 0.5 *μ*L Taq DNA polymerase (2.5 U/*μ*L, Tiagen). The assays were performed in triplicate, using the following PCR program: initial denaturation for 3 min at 94°C; 30 cycles each for denaturation for 30 s at 95°C, annealing for 45 s at 57°C; and extension for 3.5 min at 72°C; and final extension for 10 min at 72°C. All the primers used in this study are shown in Table 1.

### 2.4. Growth of the KLDS1.0391 Wild-Type Strain and* luxS* Mutant Strain

Cell numbers of the KLDS1.0391 wild-type strain and* luxS* mutant strain during growth were determined as follows: cultures, which were activated well, were inoculated in MRS broth at 2% and then incubated at 37°C for 24 h. The cultures were removed every 2 h for determining cell numbers by plate counting. The morphological features of the KLDS1.0391* luxS* mutant and wild-type strains were analyzed by scanning electron microscopy (S-3400N; Hitachi, Japan).

### 2.5. Measurement of AI-2 Activity

The AI-2 activity of the* L. plantarum* KLDS1.0391 wild-type strain and* luxS* mutant strain was determined according to the method described by Man et al. [28].

### 2.6. Acid and Bile Salt Tolerance

The KLDS1.0391 wild-type strain and* luxS* mutant strain were inoculated (2%) into MRS and MRS containing Cm, respectively, cultivated (37°C, 16 h), centrifuged (8000 ×g, 4°C, 10 min), and washed twice with phosphate-buffered saline (PBS) buffer (pH 7.2).

The acid tolerance of the mutant and wild-type strains was determined by incubation in MRS with different pH values (2.0 and 3.0). The washed cells were resuspended in fresh MRS broth with pH adjusted to 2.0 and 3.0, respectively, with HCl (1.0 mol/L). The suspensions were incubated at 37°C and then removed at 0 and 0.5 h for pH 2.0 and at 0, 1, 2, 3, and 4 h for pH 3.0. The samples were plated onto MRS agar for colony counting.

Bile salt tolerance was determined according to the method reported by Prins et al. [30], with some modifications. The washed cells were resuspended in fresh MRS broth wherein the final concentrations of bile salt (Sigma) were 0.3%, 0.5%, 1.0%, and 2.0% (w/v), respectively. Their survival counts were calculated at intervals of 2 h during an incubation period of 8 h.

### 2.7. Transit Tolerance in SDJ

#### 2.7.1. Preparation of SDJ

The SDJ was prepared according to the method reported by Barmpalia-Davis et al. [31], with some modifications. The mixture of artificial saliva (pH 6.9) contained 6.2 g/L NaCl, 2.2 g/L KCl, 1.2 g/L NaHCO_3_, and 0.22 g/L CaCl_2_. After it was autoclaved (121°C, 20 min) and cooled (to ~25°C), 3.0 g/L *α*-amylase (Sigma) was added to it and the mixture was filtered through a 0.22-*μ*m filter before use.

The simulated gastric fluid (pH 2.1) was formulated with 3.0 g/L NaCl, 1.1 g/L KCl, 0.6 g/L NaHCO_3_, and 0.15 g/L CaCl_2_. The solution was autoclaved (121°C, 20 min) and cooled (to ~25°C), followed by addition of 3.0 g/L pepsin (Sigma). Then, the mixture was filter-sterilized as described above.

Artificial intestinal fluid was prepared by suspending 5.0 g NaCl, 1.1 g KCl, and 0.3 g CaCl_2_ in 1 L distilled water. One-gram pancreatin, 0.1 g lipase, and 3 g oxgall (Sigma) were added to the solution after autoclaving and cooling. Then, the mixture was filter-sterilized as described above.

#### 2.7.2. Transit Tolerance in SDJ

To initiate the simulated digestion, the culture of the wild-type and* luxS* mutant strains (30 mL) was centrifuged (8000 ×g, 4°C, 5 min) after overnight incubation and washed twice with PBS buffer (pH 7.2). The assay was performed as follows (incubation temperature, 37°C; agitation, 50 r/min). The washed cells were added to 30 mL simulated saliva and incubated for 5 min. Afterwards, 60 mL gastric juice was added, and the pH of the mixture was maintained at approximately 3.5; the mixture was incubated for 2 h. Finally, 60 mL artificial intestinal fluid was added, and the pH of the mixture was maintained at approximately 7.5; this was followed by incubation for 4 h. The cells obtained after each incubation step were harvested (8,000 ×g, 4°C, 10 min) and washed twice with PBS (pH 7.2), following which plate counting was performed.

### 2.8. Caco-2 Cell Culture and Adherence Assay

The Caco-2 cell line was purchased from the Institute of Biochemistry and Cell Biology (SIBS, CAS, China). Caco-2 cell culture was performed using the method reported by Fernández et al. [32], with modifications. Briefly, Caco-2 cells were inoculated into Dulbecco's modified Eagle's medium (DMEM) with 10% fetal bovine serum and 1% (v/v) antibiotics (100 U/mL penicillin sodium, 1.0 *μ*g/mL streptomycin) and incubated at 37°C (5% CO_2_). The culture medium was replaced the next day. After 15–18 days, the Caco-2 cells were transferred into six-well plates and grown to confluence. A monolayer of Caco-2 cells (5 × 10^5^ CFU/mL/cm^2^) was used for adhesion assays after washing twice with PBS (pH 7.2).

The wild-type and mutant strains of KLDS1.0391 were routinely grown overnight in MRS broth. Then, the cells were collected (8000 ×g, 4°C, 10 min), washed twice with PBS (pH 7.2), and resuspended in DMEM to the final concentration of 10^8^ CFU/mL. The suspensions (5 mL) were added to the abovementioned wells containing Caco-2 cells and incubated for 2 h (37°C, 5% CO_2_). At the end of the incubation period, the Caco-2 cell cultures were washed twice with prewarmed PBS (37°C, pH 7.2) to remove the nonadherent cells and then treated with Triton X100 (1%, 10 min) to release the adhered bacterial cells. The adhesion ratio of bacterial cells was calculated by comparing the viable count on MRS agar plates before and after adhesion.

### 2.9. Inhibition of Adherence of* E. coli *ATCC 25922 and* Salmonella typhimurium* ATCC 14028 to Caco-2 Cells by* L. plantarum*

The inhibition of adhesion of pathogens to Caco-2 cells by KLDS1.0391 was performed according to previously reported methods [33–35], with some modifications. The cells of KLDS1.0391 (wild-type strain and mutant strain, hereafter referred to as “tested bacteria” in this subsection) and pathogenic strains (*E. coli* ATCC 25922 and* Salmonella typhimurium* ATCC 14028, hereafter referred to as “indicator bacteria” in this subsection) were harvested (8,000 ×g, 4°C, 10 min), washed twice with PBS (pH 7.2), and resuspended in DMEM. The cell density was adjusted to 10^8^ CFU/mL. Three different procedures, that is, competition, exclusion, and displacement, were performed to evaluate the ability of the mutant and the wild-type strains to inhibit adhesion by pathogens.

In competition assays, the suspensions of tested bacteria and indicator bacteria were added at the same time to six wells containing Caco-2 cells and coincubated for 2 h at 37°C. In exclusion assays, the suspension of tested bacteria was first added to six wells containing Caco-2 cells, incubated for 1 h at 37°C, and washed twice with 1 mL PBS to remove unadhered bacterial cells. Afterwards, indicator bacteria were added and incubated for an additional 1 h under the same conditions; the ratio of tested bacteria to indicator bacteria was 1 : 1 (v : v). In displacement assays, the order of addition of the suspensions of indicator bacteria and tested bacteria was the opposite of that used in exclusion adhesion assays. However, the other steps were the same as those for the exclusion assays. After the above steps, the Caco-2 cells were washed five times with 1 mL PBS; then, 0.5 mL 0.05% (v/v) Triton X-100 was added to each well and incubated at 37°C for 5 min to release the adhered cells. After serial dilutions till 10^−8^ with physiological saline, the numbers of viable adhering* E. coli* ATCC 25922 and* Salmonella typhimurium* ATCC 14028 cells were determined by plate counting on nutrient broth agar. The ability to inhibit adhesion was calculated as the difference between the adhesion of the pathogen in the presence and absence of* L. plantarum*.

### 2.10. Statistical Analysis

Statistical analysis was performed using the SPSS 20.0 software and* p* values (0.05) were considered statistically significant. Data were subjected to one-way analysis of variance (ANOVA). The results have been expressed as the average of three independent experiments.

## 3. Results and Discussion

### 3.1. Bioinformatics Analysis of the LuxS Protein

**Figure 1:**
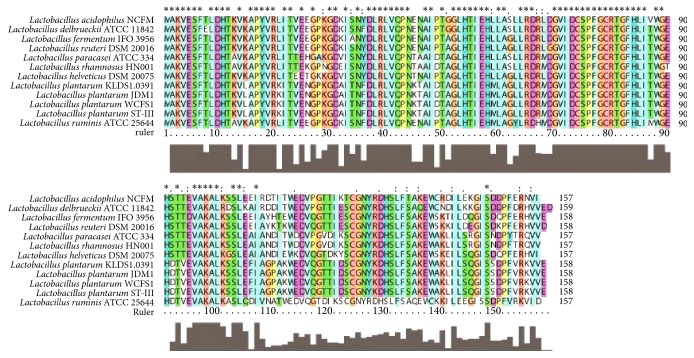
Multiple alignment sequence analysis of LuxS proteins. “*∗*” indicates conserved domains.

**Figure 2:**
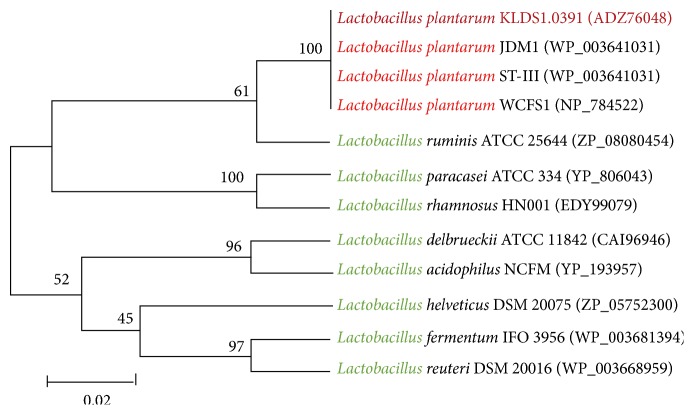
Phylogenetic tree analysis of LuxS proteins.

The presence of the* luxS* gene had been detected earlier by single oligonucleotide nested PCR (SON-PCR) [36]. The* luxS *gene was 477 bp long (GenBank accession number HQ704889) and encoded 158 amino acids. Sequence analysis suggested that the LuxS protein was located in the cytoplasm, instead of the membrane. The predicted results suggested that the LuxS protein contains one conserved region, that is, the LuxS superfamily domain (Figure S2). The LuxS superfamily is comprised of the LuxS protein, which is involved in the synthesis of the quorum-sensing signal molecule AI-2. Multiple-sequence alignment revealed that the LuxS protein of KLDS1.0391 had 100% similarity with that of* L. plantarum* WCFS1,* L. plantarum* JDM1, and* L. plantarum* ST-Ш and 77%–82% similarity with that from other reported* Lactobacillus* species (Figure 1). Phylogeny analysis showed that all the sequences formed two subfamilies. One subfamily was formed by the LuxS of* L. plantarum* KLDS1.0391,* L. plantarum* WCFS1,* L. plantarum* JDM1,* L. plantarum* ST-Ш,* L. ruminis* ATCC 25644,* L. paracasei* ATCC 334, and* L. rhamnosus* HN001. Another subfamily was formed by the LuxS of* L. acidophilus* NCFM,* L. delbrueckii *ATCC 11842,* L. helveticus* DSM 20075,* L. fermentum* IFO3956, and* L. reuteri* DSM 20016 (Figure 2). Thus, this result revealed that the LuxS of* L. plantarum* KLDS1.0391,* L. plantarum* WCFS1,* L. plantarum* JDM1,* L. plantarum* ST-Ш,* L. ruminis* ATCC 25644,* L. paracasei* ATCC 334, and* L. rhamnosus* HN001 differed from that of the other strains but that these proteins may be derived from a common ancestor. The tertiary structure of the LuxS protein in KLDS1.0391, predicted using the SWISS-MODEL software, had 48.32% similarity with the LuxS protein (*S*-ribosylhomocysteinase) of* Deinococcus radiodurans*, determined using the X-ray diffraction method (Figure S3) [37].

### 3.2. Construction of the KLDS1.0391* luxS* Mutant Strain

**Figure 3:**
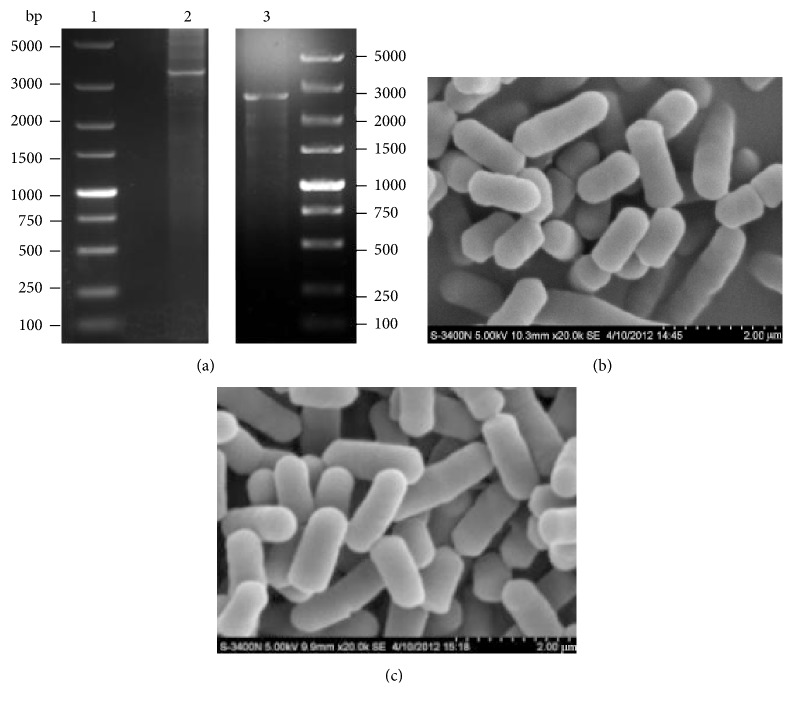
PCR products of the* luxS* gene and its flanking regions (a) and morphological features of the* Lactobacillus plantarum* KLDS1.0391* luxS* mutant (b) and wild-type strain (c) (×20,000). Lane 1: DL5000 DNA marker. Lane 2:* luxS* mutant strain. Lane 3: wild-type strain. The predicted sizes are as follows: flanking regions and the* luxS* gene of the wild-type strain, 2900 bp; the* luxS* deletion mutant, 3400 bp.

The pNZ5319 plasmid was electrotransformed into KLDS1.0391. The* luxS* deletion strain was identified by PCR (Figure 3(a)) and confirmed by sequencing (Supplementary Information-Appendix A and Appendix B). Moreover, the* luxS* deletion strain, which was cultivated for 20 generations, still did not yield a* luxS* gene band. PCR identification of the* luxS*-knockout strain showed that the homologous recombination fragment had been successfully recombined with the KLDS1.0391 genome and that part of the sequence of the* luxS* gene had been replaced by a Cm cassette. Scanning electron microscopy revealed that there was no significant difference between the morphological features of the* luxS* deletion strain (Figure 3(b)) and the wild-type strain (Figure 3(c)). Both of them appeared as short rods with uniform thickness and blunt ends, approximately 1.0–3.0 *μ*m in length and approximately 0.5–0.7 *μ*m in width.

### 3.3. Comparison of Cell Number and AI-2 Activity of the KLDS1.0391* luxS* Mutant and Wild-Type Strain during Growth at 37°C

**Figure 4:**
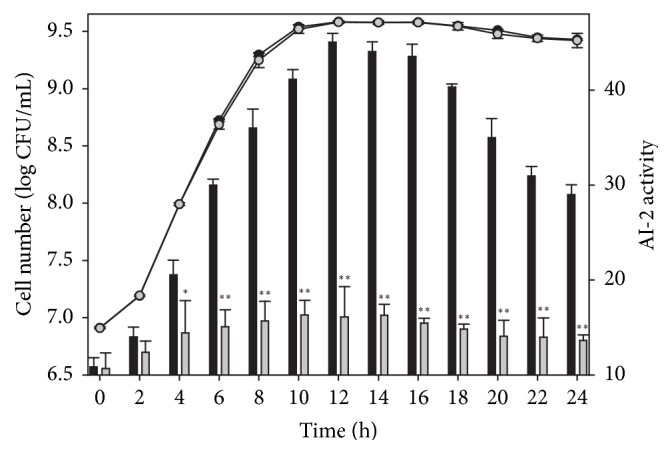
Cell number (line) and AI-2 activity (column) of* L. plantarum* KLDS1.0391 wild-type strain (black circle and black bar) and* luxS* mutant strain (grey circle and grey bar) during growth in a 24-h period in MRS at 37°C. Cell number and AI-2 activity have been expressed in terms of mean ± SD (*n* = 3). ^*∗*^*p* < 0.05 and ^*∗∗*^*p* < 0.01.

The viable cell number of the* luxS*-knockout strain did not differ significantly from that of the wild-type strain during growth at 37°C (*p* > 0.05; Figure 4). The AI-2 activity of the* luxS*-knockout strain was significantly lower than that of the wild-type strain (*p* < 0.05) during growth at 4–24 h, and only weak and stable AI-2 activity could be detected for the* luxS *deletion strain. The result suggested that the* luxS* gene is associated with AI-2 synthesis and does not affect the growth of KLDS1.0391.

### 3.4. Comparison of Tolerance of the KLDS1.0391* luxS* Mutant and Wild-Type Strains

**Figure 5:**
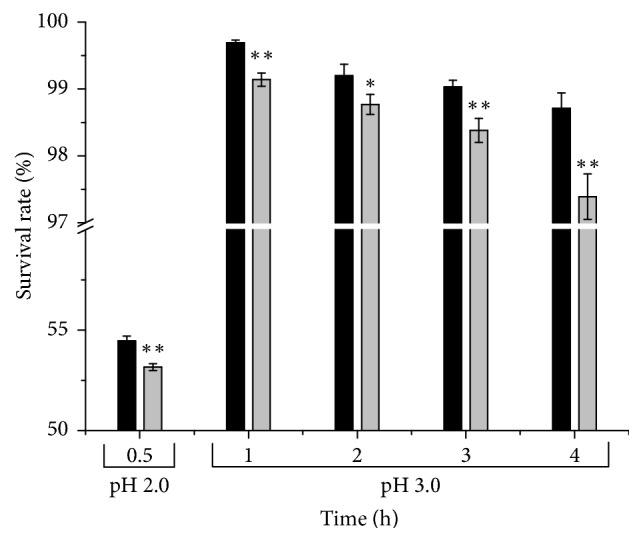
Survival of* L. plantarum* KLDS1.0391 wild-type strain (black bar) and* luxS *mutant strain (grey bar) when resuspended in acidified MRS (pH 2.0 and 3.0) at 37°C. The survival rate has been expressed in terms of mean ± SD (*n* = 3). ^*∗*^*p* < 0.05 and ^*∗∗*^*p* < 0.01.

pH values of 2.0 and 3.0 were selected because oral ingestion is the common approach for consuming* L. plantarum *[38] and the pH of the human gastric environment fluctuates between 1 and 5 during digestion. The survival rate of the* luxS *mutant strain was 53.16% after incubation at pH 2.0 for 2 h, which was significantly lower than that of the wild-type strain (54.47%; *p* < 0.01). The survival rates of the* luxS*-knockout strain and wild-type strain were all greater than 90% even after incubation at pH 3.0 for 4 h. During incubation of 0–4 h at pH 3.0, the survival rate of the mutant strain was markedly (*p* < 0.05) lower than that of the wild-type strain throughout, and the difference in survival rates between the mutant and wild-type strains was at maximum at 4 h (Figure 5).

**Figure 6:**
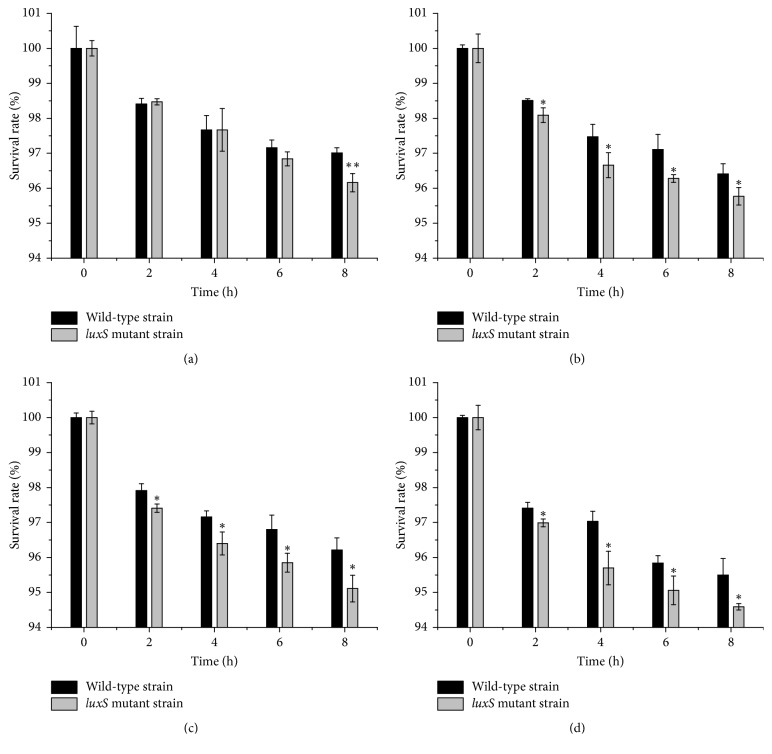
Survival of the* L. plantarum* KLDS1.0391 wild-type strain (black bar) and the* luxS* mutant strain (grey bar) when resuspended in MRS with bile salt (0.3% (a), 0.5% (b), 1.0% (c), and 2.0% (d)) at 37°C. The survival rate has been expressed in terms of mean ± SD (*n* = 3). ^*∗*^*p* < 0.05 and ^*∗∗*^*p* < 0.01.

Bile salt concentrations of 0.3–2.0% were chosen to simulate the bile salt concentration in the human GIT, in which the bile salt concentration is usually between 0.05% and 2.0%. The survival rate of the mutant was lower than that of the wild-type strain during incubation in MRS broth containing bile salt at 37°C (Figure 6). The decrease in the survival rates of the KLDS1.0391* luxS* deletion and wild-type strains became significant at 8 h in MRS broth containing 0.3% bile salt (*p* < 0.01). The survival rates of mutant and wild-type strains were 96.16% and 97.01%, respectively, at this time point (Figure 6(a)). The survival rate of the knockout strain was significantly lower than that of the wild-type strain when the concentration of bile salt was 0.5%, 1.0%, or 2.0% in MRS (*p* < 0.05 for all; Figures 6(b), 6(c), and 6(d)).

**Figure 7:**
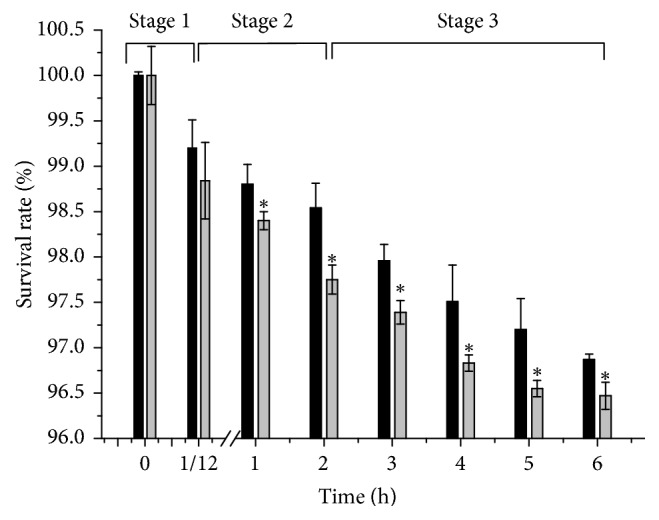
Transit tolerance of the* L. plantarum* KLDS1.0391 wild-type strain (black bar) and the* luxS* mutant strain (grey bar) in simulated digestive juice. The tolerance ability has been expressed in terms of mean ± SD (*n* = 3). ^*∗*^*p* < 0.05. Stage 1, Stage 2, and Stage 3 were digested in saliva, saliva + gastric juice, and saliva + gastric juice + gastrointestinal tract, respectively.

The transit tolerance of KLDS1.0391 in SDJ has been shown in Figure 7. The survival rates of the KLDS1.0391 wild-type strain and* luxS* mutant strain were 99.20% and 98.84%, respectively, after the two strains were incubated in artificial saliva at 37°C for 5 min; this difference was not significant. Then, SDJ was added and the mixture was incubated for 1 h; the survival rates of the mutant and wild-type strains were 98.40% and 98.80%, respectively, at this time point. After incubation for 2 h, the survival rates of the mutant and wild-type strains decreased to 97.75% and 98.54%, respectively. Moreover, the survival rate of the mutant strain showed an obvious decrease (*p* < 0.05) compared with that of the wild-type strain in a simulated gastric fluid assay. After simulated intestinal fluid was added, the survival rate of the mutant strain was significantly lower than that of the wild-type strain (*p* < 0.05) and reached 96.47% at the end of incubation for 4 h.

The tolerance of KLDS1.0391 to acid and bile salt considerably decreased and AI-2 synthesis also obviously decreased when the* luxS* gene was inactivated. The results showed that the* luxS* gene plays an important role in the acid and bile tolerance of KLDS1.0391 and is related to the bioactivity of AI-2. Furthermore, loss of the* luxS* gene weakened the tolerance of KLDS1.0391 in the SGJ. The relationship between* luxS* and acid tolerance has been confirmed in* L. acidophilus* NCFM and* L. rhamnosus* GG [17]. It was found that the transcription level trend of* luxS* was the same as that of the bioactivity of AI-2; further, the bioactivity of AI-2 of* L. acidophilus* NCFM and* L. rhamnosus* GG increased significantly in an acidic environment. Azcárate-Peril et al. [18] found that a mutant strain of* L. acidophilus* NCFM, which had a two-component regulatory system, was more sensitive than the wild-type strain to acid and that* luxS* expression was upregulated when* L. acidophilus* NCFM was in an acid stress environment. The expression of genes related to acid stress in* Streptococcus mutans *UA159 is affected by the inactivation of the* luxS* gene [39]. The survival rate of a* L. rhamnosus* GG* luxS*-knockout strain was found to decrease after incubation in gastric juice or after intake by mice; the viable count of the mutant strain in feces was only 10% that of the wild-type strain [19]. The competence of the* L. reuteri *100-23C* luxS*-deficient strain is significantly lower than that of the* L. reuteri *100-23C wild-type strain in the cecum [40].

We speculated that* Lactobacillus* could enhance the activity of AI-2, which is the quorum-sensing signal molecule, by raising the transcriptional level of* luxS* in harsh environments. This further indicated that the AI-2/LuxS quorum-sensing (QS) system could be involved in the adaptation of KLDS1.0391 to environmental factors. The existence of the AI-2/LuxS QS system in the bacteria could help them survive in the GIT. However, the phenotype of* luxS* mutant strains is pleiotropic and cannot be ascribed to QS-related signaling [39]. At the transcriptional level, Sztajer et al. [39] found that* aguA* was strongly downregulated (−73.0-fold) in* Streptococcus mutans* UA159 after deleted* luxS*.* AguA* encodes the agmatine deiminase enzyme that is used for producing ammonia to reduce the intracellular pH of less acid tolerant streptococci [41]. Notably, according to complete genome sequence, we found that the genome of KLDS1.0391 consists of several encoding proteins involved in acid and bile stress, including sodium-proton antiporters, alkaline shock proteins, F0F1-ATPase, choloylglycine hydrolase, and bile salt hydrolase [42], but not agmatine deiminase enzyme. Furthermore, the effects of the* luxS*/AI-2 QS system on the tolerance mechanisms of KLDS1.0391 at the transcriptional level and protein level require further study.

### 3.5. Capacity of Adhesion to Caco-2 Cells

The adhesion rate of the KLDS1.0391* luxS*-knockout strain (71.12%) was significantly lower than that of the KLDS1.0391 wild-type strain (82.02%; *p* < 0.05). This finding has also been noted in* L. acidophilus *[20] and* Streptococcus suis *[21]. The competitive adhesion ability of* L. reuteri* 100-23C has also been found to significantly decrease in the appendix after loss of one* luxS* gene [39]. Some researches have been performed on the relationship between* luxS* and the adhesion ability of* Lactobacillus* and* Streptococcus* in the intestinal tract [21]. AI-2 synthesis in KLDS1.0391,* L. acidophilus*, and* Streptococcus suis* was found to significantly decrease after* luxS* gene deletion or even disappear, whereas inactivation of* luxS* did not influence the growth of KLDS1.0391. Therefore, it could be speculated that the* luxS* gene played an important role in the adhesion of KLDS1.0391 to intestinal epithelial cells because of its capacity for AI-2 synthesis, instead of growth ability.

The mutant and wild-type strains of KLDS1.0391 both had an inhibitory effect on the adhesion of* E. coli *ATCC 25922 and* Salmonella typhimurium* ATCC 14028 (Table 2). The ability of the KLDS1.0391* luxS* deletion strain to inhibit adhesion to* E. coli *ATCC 25922 was significantly lower than that of the KLDS1.0391 wild-type strain (*p* < 0.05). Specifically, in the competitive, exclusion, and displacement assays, the adhesion inhibition rates of the KLDS1.0391* luxS* mutant strain decreased 7.68%, 11.9%, and 5.43%, respectively, compared to those of the wild-type strain. The ability of the KLDS1.0391* luxS*-knockout strain to inhibit competition and exclusion adhesion to* Salmonella typhimurium* ATCC 14028 was also lower than that of the KLDS1.0391 wild-type strain; however, these differences were not significant (*p* > 0.05 for both). The ability of the KLDS1.0391* luxS* deletion strain to inhibit displacement adhesion to* Salmonella typhimurium *ATCC 14028 also decreased significantly (*p* < 0.05). Overall, the ability of the KLDS1.0391* luxS* deletion strain to inhibit adhesion to* E. coli*, including inhibition of competitive, exclusion, and displacement adhesion, was significantly lower than that of the wild-type strain. However, in the case of adhesion to* Salmonella typhimurium* ATCC 14028, only the decrease in ability to inhibit displacement adhesion was significant (*p* < 0.05). To date, very little is known about the complex mechanisms of inhibition of pathogen adhesion by* L. plantarum*. The difference in the ability of KLDS1.0391 to inhibit adhesion of different pathogens may be due to the different mechanisms of adhesion. The inhibition of adhesion of KLDS1.0391 to* E. coli *may be due to the binding of surface proteins to the mannose receptor on intestinal epithelial cells. However, steric hindrance also plays a very important role in the adhesion of* Salmonella typhimurium* [35]. These results only indicated that the* luxS* genes play a role in inhibiting the adhesion of pathogenic microorganisms. The specific mechanisms involved in inhibition of adhesion of KLDS1.0391 to different pathogens still require investigation.

## 4. Conclusions

This study indicated the role of the* luxS* gene in the stress tolerance and adhesion abilities of* L. plantarum* KLDS1.0391. Initially, we found that the LuxS protein is a cytoplasmic non-transmembrane protein. Additionally, the* luxS* mutant of KLDS1.0391 was successfully constructed. Furthermore, the AI-2 activity of KLDS1.0391 markedly reduced during the 4–24 h phase of growth after deleting the* luxS* gene, but the growth of KLDS1.0391 was not influenced. In in vitro assays, deletion of the* luxS* gene had a significant effect on acid tolerance, and the same effect was obtained in the bile salt and SDJ tests. Adhesion and ability to inhibit adhesion decreased to some extent. The results indicated that the* luxS* gene was closely involved in certain probiotic properties of KLDS1.0391, which could be due to the role of* luxS *in the biosynthetic pathway of AI-2. Therefore, comprehensive analysis of the role played by* luxS* in the mechanism underlying the probiotic properties of* L. plantarum* would be of great interest.

## Figures and Tables

**Table 1:** Primer sequences.

Primer name	Sequence (5′-3′)	Annealing temperature (°C)	Function
luxS-L-f	CCGCTCGAGCGGTTTATCCCACT	57	Amplification of *luxS *left-flanking gene
luxS-L-r	AGCTTTGTTTAAACATTGCCCGTTATT		

luxS-R-f	GAGCTCGGTGTACGCAAAGTCGT	63	Amplification of* luxS *right-flanking gene
luxS-R-r	GGAAGATCTAATTCCATGTTCACCAGC		

pNZ5319-L-f	GAGCAGAATGTCCGAGAC	55	Identification of double-digested products
pNZ5319-L-r	CGGCTAAAACGACCTTAA		

pNZ5319-luxS-f	TGTTGCCGATTCCGCTAG	55	Identification of double-digested products
pNZ5319-luxS-r	ACCCCGTCAGCTTTAGG		

luxSqs-f	GTGAAAACGGTGGTGAGGTC	60	Identification of *luxS *gene mutant
luxSqs-r	TCTTTATGTGCTTTGAGCAATA		

**Table 2:** Inhibition of adherenceof *Escherichia coli *ATCC 25922 and *Salmonella typhimurium* ATCC 14028 to Caco-2 cells by the *Lactobacillus plantarum *KLDS1.0391 mutant and wild-type strains.

Strain	Competitive adhesion		Exclusion adhesion		Displacement adhesion	
	*E. coli *ATCC25922	*Salmonella*	*E. coli *ATCC25922	*Salmonella *	*E. coli *ATCC25922	*Salmonella *
		*typhimurium*		*typhimurium *		*typhimurium *
		ATCC 14028		ATCC 14028		ATCC 14028
*luxS* mutant strain	57.56 ± 3.49^b^	55.61 ± 3.56	37.80 ± 1.83^a^	65.16 ± 1.91	43.29 ± 3.17^b^	64.48 ± 1.78^b^
Wild-type strain	65.24 ± 1.93	58.30 ± 2.33	49.70 ± 1.56	67.31 ± 1.50	48.72 ± 0.69	70.40 ± 1.32

Values have been expressed as mean ± SD (*n* = 3); ^a,b^mean ± SD (%): ^a^*p* < 0.01; ^b^*p* < 0.05.

## Supplementary Materials

Supplementary Materials Figure S1: technology road map of construction of homologous recombination vector. Figure S2: conserved domain analysis of LuxS protein. Figure S3: tertiary structure of LuxS protein in* L. plantarum* KLDS1.0391 (a) and* Deinococcus radiodurans* (b). Appendix A: the luxS gene and its flank sequence of* L. plantarum* KLDS1.0391 luxS mutant strain. Appendix B: the luxS gene and its flank sequence of* L. plantarum* KLDS1.0391 wild strain.
