# Supplementary material for: Role of luxS in Stress Tolerance and Adhesion Ability in Lactobacillus plantarum KLDS1.0391
Source: Biomed Res Int. 2018 Jan 30;2018:4506829. doi: 10.1155/2018/4506829 (PMC5832066; doi:10.1155/2018/4506829)
Supplement: Supplementary Materials — Figure S1: technology road map of construction of homologous recombination vector. Figure S2: conserved domain analysis of LuxS protein. Figure S3: tertiary structure of LuxS protein in L. plantarum KLDS1.0391 (a) and Deinococcus radiodurans (b). Appendix A: the luxS gene and its flank sequence of L. plantarum KLDS1.0391 luxS mutant strain. Appendix B: the luxS gene and its flank sequence of L. plantarum KLDS1.0391 wild strain. [file 4506829.f1.doc]

Role of *luxS* in resistance to stress and adhesion ability in

*Lactobacillus plantarum* KLDS1.0391

Fang-Fang Jia, Hui-Qi Zheng, Si-Rui Sun, Xue-Hui Pang, Yu Liang, Jia-Cui Shang, Zong-Tao Zhu, Xiang-Chen Meng*

Key Laboratory of Dairy Science, Ministry of Education, Northeast Agricultural University, Harbin, 150030, China

*Corresponding author. Tel.: +86-451-55191813. Fax: +86-451-55190340.

*E-mail address*: [xchmeng@hotmail.com](mailto:xchmeng@hotmail.com) (X. C. Meng)

Figure Captions

Figure S1 Technology road map of construction of homologous recombination vector.

Figure S2 Conserved domain analysis of LuxS protein.

Figure S3 Tertiary structure of LuxS protein in *L. plantarum* KLDS1.0391 (a) and *Deinococcus radiodurans* (b).

Figure S1


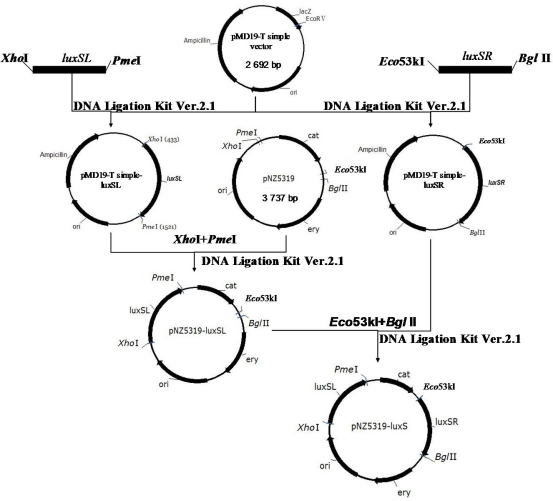


Figure S2


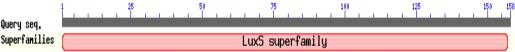


Figure S3


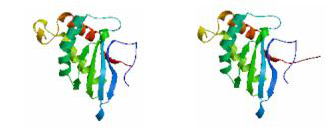


a

b

Appendix A: The *luxS* gene and its flank sequence of

*L. plantarum* KLDS1.0391 *luxS* mutant strain

TCTTTATGTGCTTTGAGCAATACCGTTGCAGCTGGTGCTTCGTAAACTTCCCGTGACTTGATACCGACCAACCGGTTTTCAATATGGTCGATCCGACCAATTCCATGTTCACCAGCAATCTGATCCAACTTGATGATTAAATCAGCTAGGTTAAGTGATTCACCATCTAAAGCAACTGGCACCCCAGCTTCAAAAGTGATTTCCAAAGTCGTTGGCGTATCCGGCGTATCAGCTAAGGCCTTCGTCCGGTCAAACGCATCGGCAGGCGCGCCCTGCCATGGATCTTCTAGAATGCCACATTCATTGGCCCGGCCCCATAAGTTTTCGTCAATCGAGTAAGGACTGTCCAAATTGATTGGTAC

GGGAATATTGTGTTCCTTAGCGTAATCGATCTCTTCTTCCCGTGACCAGTGCCAATCACGTACAGGCGCTTCGATCTTGATATCCGGTGCAAGAGCGTGGATAGCAACCTCAAACCGAACCTGATCATTACCCTTCCCAGTACAACCATGGGCAATTGCAACAGCGTGCTCTTGCTTGGCTAATGTGACTAACTTCTTAGCAATTAAAGGACGTGAAAGTGCTGAGACCAGCGGATATTCACCTTCATACAAGGTATGACCTTGTAAAGCAATTAACGCATATTCTTCAGCAAATTCTTGCTTAGCATCAATCATATATGATGAAACAGCCCCTAATTTTAGGGCCTTTTCTTTGATTGCATCCATGTCCTTGCCTTCACCAACATCAATTCCGCAGGCCACAACATCATAGCCCTTATCCTTCAACCAACTAATTGCGACCGACGTATCTAAACCACCAGAATAAGCGAGAATAATTTTATCATTTTGTTTGACCATTGATTTGTACCTCCGAAATTTTTATCTTTGCTTTTATCAATGACAGTATCGTACCACCCAATTTAATTTTATTCAATAATATTTTAAATATATTCATCATTTGTGCAATATTCACTAAAATGTAGAATTTTTATGCAGACATTCACCGAAATCAGTATATTTTTTGTTATTTTTGAAACACTACCTTAATCTAATCTTAGATTGTTTTCCCACGATTCAGGACCGCTAAGCTCGCACAATGTCAGTAAACAGCAAAAAGAGCCTAGCTTTTCGTTATGAAAAGCTAGGCTCTTTTTAATTGGTGGCCGGCACCACCATTACTTTTTATATTTTGGTCTATTCAACGACTTTGCGTACACCGAGCTCATGCCCGGGCTGTACCGTTCGTATAGCATACATTATACGAAGTTATGGGTCCCTAGCGGAATCGGCAACAGTTTAACGATTACTTCGGTAATGATTCCTAACGTGCCTTCCGAACCAATCATCAATTGCGTTTAGGATCCAAGTACAGTCGGCATTATCTCATATTATAAAAGCCAGTCATTAGGCCTATCTGACAATTCCTGAATAGAGTTCATAAACAATCCTGCATGATAACCATCACAAACAGAATGATGTACCTGTAAAGATAGCGGTAAATATATTGAATTACCTTTATTAATGAATTTTCCTGCTGTAATAATGGGTAGAAGGTAATTACTATTATTATTGATATTTAAGTTAAACCCAGTAAATGAAGTCCATGGAATAATAGAAAGAGAAAAAGCATTTTCAGGTATAGGTGTTTTGGGAAACAATTTCCCCGAACCATTATATTTCTCTACATCAGAAAGGTATAAATCATAAAACTCTTTGAAGTCATTCTTTACAGGAGTCCAAATACCAGAGAATGTTTTAGATACACCATCAAAAATTGTATAAAGTGGCTCTAACTTATCCCAATAACCTAACTCTCCGTCGCTATTGTAACCAGTTCTAAAAGCTGTATTTGAGTTTATCACCCTTGTCACTAAGAAAATAAATGCAGGGTAAAATTTATATCCTTCTTGTTTTATGTTTCGGTATAAAACACTAATATCAATTTCTGTGGTTATACTAAAAGTCGTTTGTTGGTTCAAATAATGATTAAATATCTCTTTTCTCTTCCAATTGTCTAAATCAATTTTATTAAAGTTCATTTGATATGCCTCCTAAATTTTTATCTACCTAGTATAGCATTTTGTGAAGTTTTTTTCTAGTCCAAGCTCACAAAAATCCAAAGTAACCGCTTTATTAAGCCATTCTTAAATAAAAATAAAAAAAGATTAATAGCTAAAACTATTAATCTTATCATATCCCGAGGACCGAATTTAAACTGCATGGTACCTTACTTAACTTCGCGACCATCGGCTAAAACGACCTTAACGCCTAAGACATTGTCATAACTTCGTATAGCATACATTATACGAACGGTAGATTTAAATTGTTTAAACATTGCCCGTTATTTAGCAGGTGCAGTTGCTTCACGTGCTCGATCCCGTTCTAGAACGGGCTTCAAGTATTGGCCAGTATAACTTTCAGCCACTTCAGCGACCTGTTCTGGTGTCCCAGTAGCAACGATTGTTCCGCCGCCGTCACCGCCTTCTGGGCCCAAGTCAATCAAATAATCCGCCGACTTCACAACATCCAAATTATGCTCGATGATTAACACGGTATTACCCGCATCCACCAACCGATCCAAGACACCAATCAAGCGCCGAATATCTTCACTGTGCAACCCAGTCGTTGGTTCGTCCAAGATGTAGAAATTCTTCCCCGACTGTTGTTTGTGCAATTCGGAAGCTAACTTCATCCGTTGGGCTTCTCCACCTGATAAAGTCGTGGCGGGTTGGCCTAACTTAACATAGCCCAAACCAACGTCGACCAAGGTCTGTAGTTTCCGTCGAATCTTTGGAATTGGTTCAAAGAACTTAACAGCTTCTGAAGCGGTCATTTGAAGAACATCGGCAATATTTTTGCCTTTGTATTCAACTTCCAACGTTTCAGAATTGTATTGCTTACCATGACACACTTCACAAGGTACAAAGACATCGGGCAAGAAATTCATTTCAATTTTTAGAATCCCGTCACCGTGACAAGCCTCACAACGACCACCCTTAGTATTAAAACTAAAGCGGCCCTTCTTGTAACCACGTAATTTAGCTTCATTCGTTTGTGCAAATAAATCACGAATATTGTCAAAGACACCAGTATATGTGGCCGGATTACTGCGTGGTGTTCGGCCGATTGGACTTTGGTCAATATTAACTAACCGTTCAATATTCTTAATACCACTGACACTCTTATACTTACCAGGCTTTTCAGAATTACGATTCAACTTCTGTGCCAATACGCGTTTCAAAACATCGTTAACCAAGGTCGACTTACCAGAACCGGAAACCCCCGTTACCACGACAAATTCGCCTAGTGGAAAATCAACGTCAATTTGCTTGAGGTTGTTTTCGGCCGCACCAGTGATACGAATCTTCTTACCATTACCCGGACGCCGAGTTTCAGGTAGTGGGATAAACCGTTTGCCCGATAGATATTGGCCGGTCAGTGACTTCCGCGAGCGAGCCACTTGCTTGGGCGTTCCGGCCGCCATGACCTCACCACCGTTTCAC

Appendix B: The *luxS* gene and its flank sequence of

*L. plantarum* KLDS1.0391 wild strain

AGCAGTGGCTCGCTCGCGGAGTCACTGACCGGCCAATATCTATCGGGCAAACGGTTTATCCCACTACCTGAAACTCGGCGTCCGGGTAATGGTAAGAAGATTCGTATCACTGGTGCGGCCGAAAACAACCTCAAGCAAATTGACGTTGATTTTCCACTAGGCGAATTTGTCGTGGTAACGGGGGTTTCCGGTTCTGGTAAGTCGACCTTGGTTAACGATGTTTTGAAACGCGTATTGGCACAGAAGTTGAATCGTAATTCTGAAAAGCCTGGTAAGTATAAGAGTGTCAGTGGTATTAAGAATATTGAACGGTTAGTTAATATTGACCAAAGTCCAATCGGCCGAACACCACGCAGTAATCCGGCCACATATACTGGTGTCTTTGACAATATTCGTGATTTATTTGCACAAACGAATGAAGCTAAATTACGTGGTTACAAGAAGGGCCGCTTTAGTTTTAATACTAAGGGTGGTCGTTGTGAGGCTTGTCACGGTGACGGGATTCTAAAAATTGAAATGAATTTCTTGCCCGATGTCTTTGTACCTTGTGAAGTGTGTCATGGTAAGCAATACAATTCTGAAACGTTGGAAGTTGAATACAAAGGCAAAAATATTGCCGATGTTCTTCAAATGACCGCTTCAGAAGCTGTTAAGTTCTTTGAACCAATTCCAAAGATTCGACGGAAACTACAGACCTTGGTCGACGTTGGTTTGGGCTATGTTAAGTTAGGCCAACCCGCCACGACTTTATCAGGTGGAGAAGCCCAACGGATGAAGTTAGCTTCCGAATTGCACAAACAACAGTCGGGGAAGAATTTCTACATCTTGGACGAACCAACGACTGGGTTGCACAGTGAAGATATTCGGCGCTTGATTGGTGTCTTGGATCGGTTGGTGGATGCGGGTAATACCGTGTTAATCATCGAGCATAATTTGGATGTTGTGAAGTCGGCGGATTATTTGATTGACTTGGGCCCAGAAGGCGGTGACGGCGGCGGAACAATCGTTGCTACTGGGACAACCAGAACAGGTCGCTGAAGTGGCTGAAAGTTATACTGGCCAATACTTGAAGCCCGTTCTAGAACGGGATCGAGCACGTGAAGCAACTGCACCTGCTAAATAACGGGCAATGTGCTACACTTGAGTTTGTAATGTAATTTTAATTTAGAGGAGTTGATTGTACATGGCTAAAGTAGAAAGTTCTACATTAGATCATACCAAGGTTTTAGCACCTTACGTTCGTAAAATTACGGTGGAAAATGGGCGTAAGGGTGATGCCATCACTAATTTTGATTTGCGGTTAGTTCAACCTAACAAGACCGCTATTGATACGGCGGGCTTACACACGATTGAACATATGTTGGCTGGGTTATTACGTGATCGTATGGATGGCGTGATAGCCTGCTCACCATTTGGTTGTCGGACTGGTTTTCATTTGATTACCTGGGGTGAACATGACACCGTTGAAGTTGATAAGGCATTGAAGTCCTCATTAGAATTCATTGCTGGCCCAGCTAAGTGGGAAGACGTACAAGGGACGACCATCGATAGCTGTGGGAATTACAAGGATCATTCTTTATTCTCAGATAAGGAATGGGCCAAGTTGATTTTATCGCAAGGAATTTCATCGGATCCATTTGTACGCAAAGTCGTTGAATAGACCAAAATATAAAAAGTAATGGTGGTGCCGGCCACCAATTAAAAAGAGCCTAGCTTTTCATAACGAAAAGCTAGGCTCTTTTTGCTGTTTACTGACATTGTGCGAGCTTAGCGGTCCTTGAATCGTGGGAAAACAATCTAAGATTAGATTAAGGTAGTGTTTCAAAAATAACAAAAAATATACTGATTTCGGTGAATGTCTGCATAAAAATTCTACATTTTAGTGAATATTGCACAAATGATGAATATATTTAAAATATTATTGAATAAAATTAAATTGGGTGGTACGATACTGTCATTGATAAAAGCAAAGATAAAAATTTCGGAGGTACAAATCAATGGTCAAACAAAATGATAAAATTATTCTCGCTTATTCTGGTGGTTTAGATACGTCGGTCGCAATTAGTTGGTTGAAGGATAAGGGCTATGATGTTGTGGCCTGCGGAAT

TGATGTTGGTGAAGGCAAGGACATGGATGCAATCAAAGAAAAGGCCCTAAAATTAGGGGCTGTTTCATCATATATGATTGATGCTAAGCAAGAATTTGCTGAAGAATATGCGTTAATTTGCTTTACAAGGTCATACCCTTGTATGAAGGTGAATATCCGCTGGTCTCAGCACTTTCACGTCCTTTAATTGCTAAGAAGTTAGTCACATTAGCCAAGCAAGAGCACGCTGTTGCAATTGCCCATGGTTGTACTGGGAAGGGTATCATGATCAGGTTCGGTTTGAGGTTGCTATCCACGCTCTTGCACCGGATATCAAGATCGAAGCGCCTGTACGTGATTGGCACTGGTCACGGGAAGAAGAGATCGATTACGCTAAGGAACACAATATTCCCGTACCAATCAATTTGGACAGTCCTTACTCGATTGACGAAAACTTATGGGGCCGGGCCAATGAATGTGGCATTCTAGAAGATCCATGGCAGGGCGCGCCTGCCGATGCGTTTGACCGGACGAAGGCCTTAGCTGATACGCCGGATACGCCAACGACTTTGGAAATCACTTTTGAAGCTGGGGTGCCAGTTGCTTTAGATGGTGAATCACTTAACCTAGCTGATTTAATCATCAAGTTGGATCAGATTGCTGGTGAACATGGAATTGGTCGGATCGACCATATTGAAAACCGGTTGGTCGGTATCAAGTCACGGAAGT
